# The Relationship of Undergraduate Major and Housing with Depression in Undergraduate Students

**DOI:** 10.7759/cureus.786

**Published:** 2016-09-18

**Authors:** Shaun Ajinkya, James F Schaus, Michael Deichen

**Affiliations:** 1 Department of Neurology, Medical University of South Carolina; 2 University of Central Florida Health Services, University of Central Florida College of Medicine

**Keywords:** college students, mental health, psychiatry, depression

## Abstract

Introduction: Past literature has shown that college undergraduates are particularly vulnerable to depression. The objective of this study is to find if certain majors and housing arrangements are associated with major depression as assessed by the Patient Health Questionnaire (PHQ-9), after adjustment for age, gender, and family history of depression.

Methods: Participants were undergraduates at a large public university that used the university health center from April 1 - November 4, 2013. Participants completed a survey which included the PHQ-2, a validated screening test for depression. Those who scored positive were asked to take the longer PHQ-9 survey to assess for major depression. Logistic regression was used to test the significance of associations between several prescribed variables (namely, college major, housing arrangement, age, gender, and family history of depression) and outcome (major depression as assessed by the PHQ-9).

Results: Of 541 students, 71 (13.1%) scored positive on the PHQ-9 for depression. Family history was significantly associated (OR 4.20, 95% CI, 2.42, 7.29) with major depressive disorder, as was a major in the College of Arts and Humanities (OR 3.84, 95% CI, 1.18, 12.46) compared to the baseline of an undecided/interdisciplinary major.

Conclusions: A major in the College of Arts and Humanities was significantly associated with major depression. This may be significant for future efforts to target mental health interventions on college campuses.

## Introduction

Major depressive disorder (hereafter referred to simply as “depression”) is a mood disorder described by the Diagnostic and Statistical Manual of Mental Disorders Text Revision (DSM-IV-TR) as “depressed mood and/or loss of interest or pleasure in life activities for at least two weeks” [1].

Past literature has shown that college undergraduates, in part due to age factors, are particularly vulnerable to depression [2-7]. The most likely age group to have depression is ages 15-24, which overlaps the traditional undergraduate ages of 18-22 [2-3]. Past surveys of self-reported levels of depression have indicated levels anywhere from _~_17% of college students [4-5] to up to 53% of the overall population of those aged 15-24 [6]. Gender is also significantly associated with depression prevalence and severity; female college students' score significantly higher on the BDI-II, a well-known scale of depression, and the American Psychiatric Association (APA) reports that women experience depression twice as often as men do [7]. Family history is also a significant predictor of mental illnesses, including depression [7].

The health implications of depression are significant. The World Health Organization (WHO) estimates that depression will be the second-leading cause of disability worldwide by 2020 [8]. A recent population-based survey of depression found a lifetime prevalence rate of 19.2% in the United States [2]. In the general population, only 20% of those individuals diagnosed with major depression seek treatment consistent with APA guidelines [9], and a 2008 survey of college students revealed that only 24.5% of college students with diagnosed depression were receiving treatment [10].

This higher prevalence of depression in undergraduates has been attributed in part to several “stressors”, such as adjusting to college life, new environment/housing arrangements, academic stress and pressure, interpersonal relationship strains, and financial pressures [5, 11]. However, there is no current research on the relationship between depression and two components of these major stressors: undergraduate major or housing arrangement.

This study seeks to answer this using the Patient Health Questionnaire 9 (PHQ-9) survey, a well-validated measure of depression [12-13] in a sample of students seeking care (for any reason) at the student health center of a large public university. The aim of this study is to ascertain if college major and campus-associated housing significantly and independently predict major depression as assessed by PHQ-9 among undergraduates at a large public university when adjusted for age, gender, and family history of depression.

## Materials and methods

### Recruitment

Undergraduates at a large southeastern public university that use the university-run student health center (SHC) for any reason were asked permission to take the PHQ-2 preliminary questionnaire. The PHQ-2 is a two-question instrument that is part of a larger college health preventive checklist, which ascertains information about age, gender, family history of a variety of illnesses including depression, current health issues, housing, and undergraduate major. The checklist was adapted from the US Preventive Services Task Force recommendations for preventive health screening in the college population, including screening for depression, high-risk drinking, smoking, obesity, hypertension, hyperlipidemia, cervical cancer, and HIV/STI testing [14].

Those who scored positively on this screen by answering affirmatively to one or both questions on the PHQ-2 (“Over the past two weeks, have you felt down, depressed or hopeless?”, “Over the past two weeks, have you felt little interest or pleasure in doing things?”) were asked to take the longer PHQ-9 survey. A score of ≥ 10 on the PHQ-9 is consideredmajor depression [12].

The PHQ-9 is a validated survey that asks the degree to which the participant has experienced the following in the last two weeks using a four point Likert scale (Nearly Every Day, More than Half the Days, Several Days, Not at All): “little interest or pleasure in doing things”; “feeling down, depressed, or hopeless”, “trouble falling asleep or sleeping too much”, “feeling tired”, “poor or excessive appetite”, “feeling bad about yourself”, “trouble concentrating on things”, “moving or speaking so slowly that others have noticed”, and “thoughts of hurting yourself”.

A target of at least 60 students who score ≥ 10 on the PHQ-9 was set. A control group size of at least 240 was selected, which could consist of either those who scored negatively on the PHQ-2 questionnaire or the PHQ-9 questionnaire. A 4:1 control to case ratio was selected to maximize power [15].

### Inclusion and exclusion criteria

Eligibility for the study was determined when the participant completed the “encounter form” upon admission to the SHC. If a participant consented to complete the encounter form and seek care at the SHC, they automatically “agree[d] that their de-identified information might be used for research purposes”. Inclusion criteria for the study included being currently enrolled at the large southeastern public university as an undergraduate. No compensation was provided for participation.

### IRB approval

This project was a “minimum risk” project that involves human participants. In accordance with this, local institution IRB approval was sought. On February 24, 2013, the IRB approved this project (approval #SBE-13-09135); in accordance with the approval, data collection began on April 1, 2013 and was completed by November 4, 2013.

### Data management and quality control

Personnel received the completed worksheets from the participants and placed them in a secure file cabinet on the premises of the SHC. The checklist was scanned into the patient’s electronic health record (routine practice at SHC), but the PHQ-9 was de-identified and placed in the secure file by the research assistant (RA) and linked by a number to checklist data.

The RA entered the PHQ-9 and checklist data into a Microsoft Access database, which was later converted into a STATA dataset. This dataset did not contain any identifying information (names, etc.) other than the ID. Only the RA and principal investigator (PI) knew the linkage of the ID to any personal health information.

### Statistical analysis

Logistic regression was used to test the significance of associations between several study variables (college major, housing choice, age, gender, and family history of depression) and outcome (major depression as assessed by the PHQ-9). A conservative estimate for logistic regression is to have 10 cases per study variable [16]. The team wished to have enough depression cases to make significant inferences, so we targeted at least 60 cases for that purpose. In addition to the five pre-specified variables (which were “forced into” a multiple logistic regression model), variables assessing cigarette usage in the past 30 days and binge drinking in the past two weeks were “introduced” into the full model and were set to be retained or removed via stepwise selection with a cutoff of p < 0.05*. *Interaction terms between gender and major, gender and housing, and housing and major were also tested. The interaction terms were introduced into the full model and retained or removed based on the principles of “purposeful selection” [17]. The study variables of the final model were assessed for multicollinearity. To do this, the condition number and variance inflation factor (VIF) were calculated; values below 30 for the condition number and 10 for the VIF were sought in order to ensure that there was not significant multicollinearity [18].

For the purposes of analysis in logistic regression, the housing arrangement was categorized as “on campus” (living in a residence hall or Greek housing) vs. “off campus” (University affiliated apartment, parent’s home, or non-affiliated apartment or home), and the college major category was categorized as “STEM” (science, technology, engineering, and math), non-STEM, or undecided/interdisciplinary. Majors in the College of Sciences requiring a BS or majors in the Colleges of Biomedical Sciences, Engineering, and Computer Sciences were considered to be STEM majors. An undecided/interdisciplinary major was used as the reference category for the logistic regression. After viewing the data, we chose to analyze college majors in the arts and humanities as a separate category due to its high prevalence of depression.

Demographics were reported for the whole population and by those scoring positive or negative for depression. Frequencies of various demographic variables were compared between the depressed and non-depressed groups by Chi-square tests or Fisher’s exact test (if a group was to have a count less than 5). The level of confidence for all statistical tests was p < 0.05. All analyses were performed using Stata 11 (College Station, TX).

### Power

This sample has an 81% power to detect an 18% difference in major frequency among those depressed and not depressed (28% of depression cases having a STEM major vs. 10% of depression cases not having a STEM major). As there is no pilot data on frequencies in these groups, this assumption represents an estimate.

## Results

In total, 558 undergraduates participated in the survey. Of those, 541 had complete surveys on the variables being assessed (age, gender, housing arrangement, major, family history of depression, cigarette use within the past 30 days, and binge drinking). Of those 541 surveys, 109 (20.1%) gave an affirmative answer to one or both of the two PHQ-2 questions and, thus, took the PHQ-9 survey. Of those 109, 71 (65.1% of those taking the PHQ-9, and 13.1% of all surveys) scored 10 or higher, signifying major depression (also referred to as PHQ-9+). Overall, 316 (58.4%) of participants were female. The mean age of all participants was 21.3 (SD 3.8, range: 17 to 49, with 79.9% between ages 18-22, inclusive). Over half (311, 57.5%) lived off campus, with 112 (20.7%) having a self-reported family history of depression. In total, 201 (37.2%) were pursuing a STEM major.

For purely descriptive purposes, the specific choices of housing among PHQ-9 positives are shown in Table 1. “Non-affiliated apartments or home” was the most popular choice, representing nearly half of those surveyed in both groups, with residence hall a distant second, representing about a quarter of both groups.

**Table 1:** Housing Choices

	PHQ-9+ (n = 71)	PHQ-9- (n = 470)
	N (%)	N (%)
Residence Hall	18 (25.3)	106 (22.6)
Greek Housing	1 (1.4)	7 (1.5)
University Affiliated Apartment	11 (15.5)	87 (18.5)
Parent's Home	9 (12.7)	47 (10.0)
Non-affiliated Apartment or Home	32 (45.1)	223 (47.5)

Table 2, which is also for purely descriptive purposes, displays the choices of a college major by group. Notable findings included the particularly high percentage of PHQ-9 positives pursuing a major in the arts and humanities (25.4% of the PHQ-9+ total vs. 6.4% of the PHQ-9- total). Engineering and computer science represented the most common major in the PHQ-9- group. Additionally, there were no positive PHQ-9 results in the education major category.

**Table 2:** Choices of Major

	PHQ-9+ (n = 71)	PHQ-9- (n = 475)
	N (%)	N (%)
Arts and Humanities	18 (25.4)	30 (6.4)
Biomedical Sciences	6 (8.5)	49 (10.4)
Business Administration	7 (9.9)	62 (13.2)
Education	0 (0.0)	30 (6.4)
Engineering & Computer Science	6 (8.5)	70 (14.9)
Health and Public Affairs	10 (14.1)	59 (12.6)
Hospitality Management	2 (2.8)	23 (4.9)
Nursing	1 (1.4)	19 (4.0)
College of Sciences, BA Program	5 (7.0)	43 (9.2)
College of Sciences, BS Program	11 (15.5)	59 (12.6)
Undergraduate Interdisciplinary Studies	3 (4.2)	13 (2.8)
Undecided	2 (2.8)	13 (2.8)

The demographics of the study population are presented in Table 3. The “major” and “housing” variables are dichotomized as described in the “Methods” section. There were no significant differences in age, gender, living in off-campus housing, having a STEM major, or self-reported participation in binge drinking between the PHQ-9+ and PHQ-9- groups. A significantly larger percentage of those in the PHQ-9+ group had a family history of depression (46.5%) or had used a cigarette in the past 30 days (18.3%) than those in the PHQ-9- group (16.8% and 8.1%, respectively, p < 0.05 in both cases).

**Table 3:** Demographics of Survey Participants *The p-value represents a t-test for age and a Chi-squared test for all other variables. SD = standard deviation; STEM = science, technology, engineering and math

	PHQ-9+ (n = 71)	PHQ-9- (n = 470)	p-value*
	N (%)	N (%)	
Age (Mean, (SD))	21.4 (4.9)	21.3 (3.6)	0.70
Female Gender	40 (56.3)	276 (58.7)	0.70
Lived Off Campus	41 (57.8)	270 (57.5)	0.96
STEM Major	23 (32.4)	178 (37.9)	0.37
Family History of Depression	33 (46.5)	79 (16.8)	<0.05
Cigarette Use in Past 30 Days	13 (18.3)	38 (8.1)	<0.05
Binge Drinking	16 (22.5)	95 (20.2)	0.65

Table 4 shows the logistic regression performed. Interaction terms between gender and dichotomized housing arrangement, gender and major, and housing and major were tested, and none were significant. After stepwise selection, five variables were used: gender, age, housing arrangement, major, and family history of depression. In light of the high level of depression in the arts/humanities categories, the “major” variable was changed to a four-category variable describing STEM majors, arts and humanities majors, all non-STEM, non-arts and humanities (NSNAH) majors, and undecided/interdisciplinary studies majors (as a referent/baseline category). No multicollinearity issues were found in the final model.

**Table 4:** Logistic Regression Analysis of Depression Predictors All odds ratios are adjusted odds ratios. STEM = science, technology, engineering and math

	OR	95% CI
Age (1-year increment)	1.004	(0.93, 1.08)
Female Gender	1.10	(0.63, 1.90)
Lived Off Campus	0.97	(0.55, 1.71)
Family History of Depression	4.20	(2.42, 7.29)
Major		
Undecided/Interdisciplinary	1.00 (reference)	-
STEM	0.80	(0.27, 2.39)
Non-STEM (other than Arts/Humanities)	0.77	(0.26, 2.29)
Arts/Humanities	3.84	(1.18, 12.46)

Increases in age, female gender, and living off campus were not significantly associated with increased odds of testing positive on the PHQ-9 scale. Having a STEM major or an NSNAH major was not associated with higher odds of a PHQ-9 positive score relative to having an undecided/interdisciplinary major, but having a major in the arts and humanities was (OR 3.84, 95% CI, 1.18, 12.46). Having a family history of depression was also associated with higher odds of depression (OR 4.20, 95% CI, 2.42, 7.29).

## Discussion

This is, to our knowledge, the first study assessing the effect of student housing arrangements and college major on the prevalence of major depression in a major university setting. Undergraduate students, who are predominantly in the age range of 18-22, represent a vulnerable population, both with regard to developing depression and their ability to seek treatment for it, and further research is needed in this area. Research suggests that identifying college students with untreated depression has the potential for significant health improvement of college students with untreated mental health problems; a majority (65%) had positive beliefs about the effectiveness of treatment [19]. Additionally, one of the “major” categories (in particular, majors in the College of Arts and Humanities) was associated with major depression compared with a reference category of an undecided/interdisciplinary major. However, our hypothesis that living off campus would predict a higher prevalence of depression was not supported by our data. 

We found that in total, 13.1% of our study population tested positive for depression on the PHQ-9. This was close to the nationwide average of 17.9% among college students ever diagnosed with major depression as found by the NCHA survey [4].

While the majority of those testing positive (56.3%) for the PHQ-9 were women, as shown in other studies [7], a similar percentage (58.7%) of those seeking care at the SHC who were not depressed were also women, so gender was not a significant predictor of increased odds of depression. Past studies have suggested that women have a higher mean number of visits to primary care clinics [20], which suggests that our comparison groups were representative of populations that seek medical care. This suggests that a population-based sample, rather than a clinic-based sample, may be more appropriate for an analysis of the role of gender in major depression.

A self-reported family history of depression was significantly associated with higher odds of a student testing positive on the PHQ-9. This has been consistently reported throughout the literature [7, 21]. In light of this strong and consistent association, it would be prudent for university health care centers to educate the student body on this while at the same time avoiding any perception of stigmatizing those who have such a family history.

Having a STEM major was not associated with PHQ-9+. Majors in the STEM category have a popular perception of being more rigorous and stressful [22], and it was our initial hypothesis that this may have an association with major depression in college undergraduates.

A key finding of our study was that students seeking a major in the College of Arts and Humanities had higher odds of being depressed than those in the baseline category (undecided/interdisciplinary major); no significant difference was found for STEM majors or non-STEM majors outside the College of Arts and Humanities. The specific programs that encompass the College of Arts and Humanities at the university surveyed include African studies, English, the Florida Interactive Entertainment Academy, history, Judaic studies, Latin American studies, Middle Eastern studies, modern languages and literatures, music, performing arts, philosophy, texts and technology, theatre, visual arts and design, women’s studies, and writing and rhetoric. As can be seen in the Sallie Mae “How America Pays for College 2013” report, these disciplines correspond with the “liberal arts” and “visual and performing arts” categories [23]. According to the report, the “visual and performing arts” majors have the lowest starting salary of any category of a major, which may be a possible contributor to stress and depression; economic uncertainty has been shown to be associated with depression and suicide attempts in past studies [24]. Also, visual and performance arts students were shown to have the lowest rates of financial planning prior to college enrollment, which may suggest another factor leading to anxiety or depression [23]. Furthermore, there is evidence suggesting that there is a higher prevalence of depression in teens with an interest in the arts [25], a finding which may carry over to the undergraduate years.

Highly creative people may experience a greater likelihood of developing certain types of psychopathology, including mood disorders [26-27]. A meta-analysis examined 29 studies investigating a possible association between creativity and mental illness, and 15 found no evidence to link creativity and mental illness, nine found positive evidence, and five were indeterminate [28]. Thus, it is unclear if students majoring in the arts and humanities have a higher predisposition to depressive symptoms independent of factors associated with the stress and rigors of college life, financial difficulties, and the uncertainties of future careers associated with this chosen academic major.

We found that there was no association between depression and a student living on or off campus, contrary to our hypothesis. The distribution of our study sample (with 57.5% living off campus) was similar to the national distribution of American college students living off campus as measured by U.S. News and World Report (62%), which suggests that our data are generalizable to the broader population [29]. However, the Sallie Mae report “How America Pays For College” reported that in 2013, 57% of families surveyed reported that their college student lived either with parents or relatives [23]. This figure is considerably higher than the 10.4% of students in our study who lived with parents. This may suggest that students who live at a parent’s home are less likely to use the SHC or that our sample was not representative of this population. The prevalence of depression specifically among students that live at home is of importance, due to the large and increasing percentage of students who live at home/with parents.

There were several important strengths to this study. This study was the first survey of major depression to take into account the college major and housing arrangement as possible risk factors. The incorporation of study questions into the usual paperwork asked to be completed by students seeking care at the SHC ensured that the survey did not constitute any additional burden upon the participant’s time than they otherwise would have expended in a routine visit to the SHC. In addition, the routine nature of the survey allowed us to interview a very large population (541 in total, with 71 positives) that allowed us a large enough sample size for inferential methods, such as logistic regression. Our survey period spanned from April to November, which gave a good representation of students during the spring, summer, and fall semesters. Moreover, the fact that the survey was set at a large, ethnically and socioeconomically diverse public university allowed for the study to be generalizable.

However, there were also several limitations to this study. The non-prospective nature of the study did not allow for a causal determination of whether any of the predictors affected depression. Our data showing an association of a major in the arts and humanities and depression did not address the root cause of this association.  Also, the fact that some of the questions had missing data (which were censored, as described in the Results), may indicate that in the future, a more robust method of data collection (such as an electronic survey that prompts the user to complete any missing information or to indicate specifically that they do not want to fill it out) should be used. The study was also conducted with the aim of getting a certain number of cases for analysis, meaning that the overall prevalence of depression may be biased. Our decision to not record a response rate was also a weakness. In addition, the study population was a convenience sample from a healthcare center, which may not represent the overall prevalence or characteristics of people with depression in the student population. Population or community-based research methods may be appropriate for further studies. Another limitation is that this study did not assess correlations of housing arrangements or college major with the severity of depression (scores > 15 or > 20 on the PHQ-9), and simply stated yes/no with a score of 10 or more. In addition, while the PHQ-9 is a well-validated screen for depression that measures the degree to which a patient suffers from nine symptoms of depression, a PHQ-9 score of 10 or more is not synonymous with a diagnosis of a major depressive disorder (MDD), which requires a thorough clinical evaluation [12].

The decision to not separately record the race/ethnicity of participants was also a weakness, as it did not allow us to consider this variable as a predictor or mediator with our other variables or to measure the generalizability of our sample. However, a “common data set” produced by the University in 2013 suggested that the overall demographics of university undergraduates, calculated from their table, was non-Hispanic White 57.1%, non-Hispanic Black or African-American 10.5%, Hispanic 21.5%, non-Hispanic Asian or Pacific Islander 5.6%, non-Hispanic Native American/Alaska Native 0.2%, non-Hispanic people of two or more races 2.9%, non-Hispanic Native Hawaiian/Pacific Islander 0.2%, and non-resident alien 1.1%, with 0.8% unknown. This suggests a diverse population using the SHC [30].

The results of this study suggest several possible areas for future research. Since each of the surveys was dated, a study into whether the time of year is associated with higher levels of depression may be of interest, as university health centers can then target interventions based on the time of year. 

## Conclusions

In conclusion, our study found that a major in the “College of Arts and Humanities” was significantly associated with higher odds of depression (as assessed by the PHQ-9), while housing arrangement was not. This study raises questions about how to best target the high-risk group of students who major in the Arts and Humanities and further investigation into the causal nature of this association, and optimal screening and early intervention services to address depression in this high-risk group of college undergraduates would be prudent. More research is needed to investigate mental health problems and health service utilization associated with academic majors at other institutions of higher education, many of which specialize in high-risk areas of academic and professional interest. 
